# Targeted therapies for myeloproliferative neoplasms

**DOI:** 10.1186/s40364-019-0166-y

**Published:** 2019-07-16

**Authors:** Bing Li, Raajit K. Rampal, Zhijian Xiao

**Affiliations:** 1 MDS and MPN Centre, Institute of Hematology and Blood Diseases Hospital, Chinese Academy of Medical Sciences & Peking Union Medical College, 288 Nanjing Road, Tianjin, 300020 China; 2 State Key Laboratory of Experimental Hematology, Institute of Hematology and Blood Diseases Hospital, Chinese Academy of Medical Sciences & Peking Union Medical College, Tianjin, China; 3 Leukemia Service, Department of Medicine, Memorial Sloan Kettering Cancer Center, New York, USA

**Keywords:** Targeting therapy, Myeloproliferative neoplasms

## Abstract

The discovery of *JAK2*V617F and the demonstration that BCR-ABL-negative myeloproliferative neoplasms (MPNs) are driven by abnormal JAK2 activation have led to advances in diagnostic algorithms, prognosis and ultimately also treatment strategies. The JAK 1/2 inhibitor ruxolitinib was a pivotal moment in the treatment of MPNs, representing the first targeted treatment in this field. Despite a weak effect on the cause of the disease itself in MPNs, ruxolitinib improves the clinical state of patients and increases survival in myelofibrosis. In parallel, other JAK inhibitors with potential for pathologic and molecular remissions, less myelosuppression, and with greater selectivity for JAK1 or JAK2, and the ability to overcome JAK inhibitor persistence are in various stages of development. Moreover, many novel classes of targeted agents continue to be investigated in efforts to build on the progress made with ruxolitinib. This article will discuss some of the advances in the targeted therapy in this field in recent years and explore in greater detail some of the most advanced emerging agents as well as those with greatest potential.

## Background

The classic *BCR-ABL1*–negative MPNs include three different disorders, essential thrombocythemia (ET), polycythemia vera (PV), and primary myelofibrosis (PMF), and are caused by constitutive activation of the cytokine receptor/JAK2 pathway due to acquired somatic mutations in three major genes [1]. *JAK2*V617F is the most prevalent mutation in MPNs associated with the three disorders (65–70%) and is present in 95% of PVs. Mutations in exon 12 of JAK2 are found in around 2% of PV, which are negative for the *JAK2*V617F mutation. Mutations in the thrombopoietin receptor (MPL) gene are mainly present in 5–10% ET and PMF. The most frequent are substitutions of the Trpytophan(W)515 residue to several other amino acids, mostly Leucine(L) or Lysine (K). The third gene found frequently mutated in MPNs is calreticulin (*CALR*), which is affected by mutations leading to a + 1 frameshift in the exon 9. As with *MPL* mutations, *CALR* mutations are associated with ET and PMF but with a higher frequency (25–35%). CALR is not a molecule directly involved in activation of JAK2, but the new C-terminus common to all mutants allows the CALR mutants to tightly bind and activate MPL and JAK2 [2–5].

With greater understanding of the key role of JAK-STAT pathway in MPN pathogenesis, the development of the JAK 1/2 inhibitor ruxolitinib was a pivotal moment in the treatment of MPN, representing the first targeted treatment in this field. Although ruxolitinib is known to have striking impacts on reduction in spleen size and reduction in symptom burden, there are still many clinical challenges encountered during the use of ruxolitinib. Therefore, novel drugs and targets are being explored as mono-or combination-therapy in this field. This article will discuss some of the advances in the targeted therapy in this field in recent years and explore in greater detail some of the most advanced emerging agents as well as those with greatest potential.

### JAK inhibitors

#### Type I inhibitors

Type I inhibitors target the ATP-binding site of the JAKs under the active conformation of the kinase domain [6]. Most clinically tested inhibitors are type I. They differ in their specificity for JAK2. Many inhibitors target both JAK2 and JAK1 (ruxolitinib and momelotinib). Less frequently, they target only JAK2 (NS-018, pacritinib and fedratinib).

##### Ruxolitinib

The oral JAK1/2 inhibitor, ruxolitinib was the first approved targeted treatment for intermediate- or high-risk myelofibrosis (MF) on the basis of the results of the Controlled Myelofibrosis Study with Oral JAK Inhibitor Treatment-I (COMFORT-I) [7] and COMFORT-II [8] clinical trials and for patients with PV who are refractory to or intolerant of hydroxyurea on the basis of the results of the Randomized Study of Efficacy and Safety in Polycythemia Vera With JAK Inhibitor INCB018424 Versus Best Supportive Care (RESPONSE) [9] clinical trial.

In COMFORT-I, 309 patients were randomized to either ruxolitinib or placebo, with a ≥ 35% reduction in spleen volume seen in 41.9% treated with ruxolitinib vs. 0.7% in the placebo group. In COMFORT-II, ruxolitinib was compared with best available therapy (BAT) in 219 patients, randomized in a 2:1 ratio. Similarly, the primary end point of a reduction in spleen size ≥35% by week 48 was seen in 28.5% of patients treated with ruxolitinib compared with 0% in the BAT group. The EORTC-QLQ-C30 scores for symptoms relevant to patients with MF showed an improvement from baseline by week 8 and continued through to week 48, indicating significant improvement in quality of life.

Following COMFORT studies, the JUMP (JAK Inhibitor RUxolitinib in Myelofibrosis Patients) study [10] was initiated. JUMP was a phase 3b expanded-access trial for patients in countries without access to ruxolitinib outside of a clinical study and included those classified as intermediate-1 risk, a population that was not included in COMFORT studies. This study further confirmed the safety and efficacy findings from an analysis of 1144 patients with intermediate- or high-risk MF, including for those patients with intermediate-1-risk disease. Furthermore, JUMP was a global study conducted in a setting that resembles routine clinical practice. Findings from this study help guide clinicians in the management of their patients with MF.

Based on COMFORT-I and COMFORT-II clinical trials, analysis of patients treated for several years with ruxolitinb indicated a significant increase in survival in Int-2 and high-risk MF, The survival benefit with ruxolitinib was observed irrespective of baseline anemia status or transfusion requirements at week 24. But progression to leukemia was not significantly different [11, 12]. It is possible that most pro-survival effects derive from its palliative anti-inflammatory effects. Further analyses will be important for exploring ruxolitinib earlier in the disease course to assess the effect on the natural history of MF.

The RESPONSE study evaluated the efficacy of ruxolitinib in PV patients who were either refractory or intolerant to hydroxyurea, and had ongoing venesection requirement and splenomegaly. Patients were randomized on a 1:1 basis between ruxolitinib and BAT with 22.7% of patients in the ruxolitinib group meeting the composite end points of HCT control and > 35% splenic volume reduction at 32 weeks, compared with 0.9% in the BAT group. In RESPONSE-2 [13], 173 PV patients again resistant or intolerant to hydroxycarbamide, but without splenomegaly, were randomized between ruxolitinib and BAT, with the primary end point of HCT control achieved in 62% in the ruxolitinib group compared with 19% treated with BAT.

In refractory or hydroxyurea-resistant ET patients, ruxolitinib offered no advantage compared with other therapies in the control of the thrombocytosis and disease complications but did alleviate general symptoms and pruritus [14]. In the other [15], which was an open-label phase 2 trial, ruxolitinib induced a meaningful reduction in platelet levels and attenuated ET-related symptoms. These preliminary results seemed superior to historically observed results, but this study was done in the absence of a comparison with another treatment.

Overall, ruxolitinib is a well-tolerated oral treatment with approximately 25–33% of adverse effects. The main toxicities are hematological, moderate anemia that may correct with time, and thrombocytopenia, which can be very severe in high-risk MF. Middle-term toxicity is an immune suppression that may be responsible for reactivation of viral infections, particularly herpes zoster and HIV1 and bacterial infections such as pneumonia, tuberculosis reactivation and urinary tract infections [16]. Long-term monitoring will be important because ruxolitinib decreases natural killer cell functions with a potential risk of solid tumor and lymphoma development [17, 18].

Despite these clinical benefits, chronic therapy with ruxolitinib has not led to molecular or pathologic remissions in the majority of MPN patients [7, 8] in contrast to ABL kinase inhibitors in chronic myeloid leukemia (CML). Moreover, hematological toxicity, which is generally ruxolitinib-dose dependent and reversible, is an issue for many patients. As such there is a significant population of PMF patients who are excluded from treatment with ruxolitinib, with most avoiding its use in patients with a platelet count of < 50 × 10^9^/l.

##### Momelotinib

Momelotinib (CYT38) is a JAK1/JAK2 inhibitor that has shown activity resembling ruxolitinib with respect to spleen size reduction and constitutional symptom alleviation [19–21]. Importantly, momelotinib was shown to ameliorate anemia, which is a major concern in MF. These promising results led to the opening of a phase III trial for the Momelotinib Versus Ruxolitinib in Subjects With Myelofibrosis (SIMPLIFY)-1 and − 2 studies in MF. However, the results of the two clinical trials did not show a major advantage of momelotinib on ruxolitinib in a ≥ 35% reduction in spleen volume at week 24, although momelotinib was associated with a decrease in transfusion requirements [22, 23].

##### Pacritinib

Pacritinib (SB1518) is a JAK2/FLT3 inhibitor. Striking results were obtained in phase 1–2 clinical trials [24]. At week 24, a ≥ 35% reduction in spleen volume determined by magnetic resonance imaging (MRI) was achieved in 23.5% of evaluable patients (4/17), a ≥ 50% spleen length reduction as measured by physical examination was achieved in 47.4% (9/19). Meanwhile, 38.9% of evaluable patients (7/18) achieved a ≥ 50% decrease in MF Quality of Life and Symptom Assessment total score. Pacritinib was administered to patients with low platelet levels, as it appeared less myelosupressive. The reasons behind this feature are unclear; they could be linked to reduced specificity for MPL/JAK2 complexes. Subsequently, two phase 3 clinical trials (PERSIST − 1 and − 2) were started with different doses of pacritinib [25, 26].

In PERSIST-1, 327 patients were randomly assigned to pacritinib 400 mg once daily (*n* = 220) or BAT excluding JAK2 inhibitors until disease progression or unacceptable toxicity (*n* = 107). At week 24, a ≥ 35% spleen length reduction was achieved by 42 (19%) patients in the pacritinib group versus five (5%) patients in the BAT group. To compare the efficacy of pacritinib with BAT, including ruxolitinib, in patients with myelofibrosis and thrombocytopenia and determine if a lower dose of pacritinib would be safer with maintained efficacy, PERSIST-2 was designed. 311 Patients were randomized 1:1:1 to pacritinib 400 mg once daily, pacritinib 200 mg twice daily, or BAT. Pacritinib twice daily led to significant improvements in a ≥ 35% spleen length reduction and 50% reduction in TSS versus BAT. Clinical improvement in hemoglobin and reduction in transfusion burden were greatest with pacritinib twice daily. Collectively, Pacritinib can provide a treatment option for patients with myelofibrosis and baseline thrombocytopenia, for whom current treatment options are limited.

##### Fedratinib

Fedratinib (TG101348) was assessed during the JAKARTA trials with interesting clinical results [27–29]. In JAKARTA-1, 289 patients with intermediate-2 or high-risk MF were randomly assigned to fedratinib or placebo for at least 6 consecutive 4-week cycles. The primary end point of a reduction in spleen size ≥35% at week 24 was achieved in 38.3% of patients in the fedratinib group, vs 1% in the placebo group. Symptom response rates at week 24 were 35.1% in the fedratinib group compared with 7% in the placebo group. To further investigate the efficacy and safety of fedratinib in patients with ruxolitinib-resistant or ruxolitinib-intolerant MF, 97 patients were enrolled and received at least one dose of fedratinib in JAKARTA-2. Of 83 assessable patients, 46 (55%) achieved a reduction in spleen size ≥35% at week 24, suggesting that patients with ruxolitinib-resistant or ruxolitinib-intolerant myelofibrosis might achieve significant clinical benefit with fedratinib. But rare patients developed Wernicke encephalopathy, which led to its stop [29]. It was assumed that it was related to an inhibition of thiamine uptake, although fedratinib does not lead to inhibition of thiamine uptake in rats [30]. The FDA removed the clinical hold in august 2017 and clinical trials are being planned.

##### Ns-018

NS-018 is a JAK2/Src inhibitor that has been assessed in patients with *JAK2*V617F-positive MF, ET, and PV. NS-018 showed an apparent increased potency for the JAK2V617F mutant in mouse models, possibly leading to less immunosuppressive effects [31]. It was tested in MF with symptom improvement but minor impact on *JAK2*V617F allele burden [32].

#### Type II inhibitors

Type II inhibitors recognize the inactive conformation of kinases and bind to the ATP-binding pocket and to the extra ‘DFG-out’ pocket only accessible in the conformation of inactive kinases. The so-called ‘DFG-out’ pocket is created by the DFG phenylalanine adopting an ‘out’ conformation, which means that its side chain is out of a hydrophobic spine found in the active conformation. By exploiting supplementary binding sites contiguous to the ATP-binding pocket (the DFG- pocket), type II inhibitors can gain in specificity and hence in selectivity [6, 33, 34]. Importantly, the extra pocket targeted by type II inhibitors is less conserved within the kinome than the canonical ATP-binding pocket, thus promising better selectivity of the compound and minimizing adverse effects due to inhibition of unintended kinases.

Two type II JAK2 inhibitors (NVP-BBT594 and NVP-CHZ868) have been developed. NVP-CHZ868 has been used in preclinical models and was very effective [35]. Both inhibitors were amenable for drug development.

### Ruxolitinib plus other agents

Despite the significant improvements that have been seen with the use of ruxolitinib in the management of patients with MF, it is recognized that it has some limitations, which include limited disease modifying activity. The COMFORT-II study showed only a 7% median reduction in JAK-2 allele burden at 48 weeks in the ruxolitinib group [36]. It is now recognized that several of the positive effects from ruxolitinib therapy may be due to a reduction in inflammatory cytokine activity rather than targeting clonal, stem cell-derived myeloproliferation, which is the primary driver of the disease. Furthermore, the median duration of spleen response is 3 years, with approximately 50% of patients discontinuing ruxolitinib treatment by 3–5 years either due to disease progression or intolerance. In view of these factors, many studies are currently evaluating the potential for combination therapy of novel agents given alongside ruxolitinib, to maximize its efficacy and further extend the duration of response.

#### Ruxolitinib+Azacitidine

Abnormalities in DNA methylation are recognized in MPN, with JAK2 mutated patients showing impaired methylation of histone subsets, due to higher affinity binding of protein arginine methyltransferase 5 (PRMT5), resulting in promotion of myeloproliferation [37] (Will be discussed in the new targeting therapy).

Encouraging preliminary results have also been recently reported from phase 1–2 trials of combination therapy with ruxolitinib and the hypomethylating agent azacitidine, in patients with MF [38, 39]. Patients were included with an overall response rate of 69%, and a > 50% palpable spleen length reduction at 24 weeks was seen in 48% of patients, higher response rates than seen with ruxolitinib monotherapy. It was also noted that 26% of the spleen reductions occurred after addition of azacitidine, adding weight to the potential synergistic effect. Interestingly, reduction in JAK2 allele burden was seen in 87% of assessable patients and a reduction in bone marrow fibrosis was also seen in 41%.

#### Ruxolitinib+ histone deacetylas (HDAC) inhibitor

HDAC inhibitors are a class of epigenetic modulators. A phase 1 study went on to investigate combination therapy with HDAC inhibitors panobinostat and ruxolitinib. The results were encouraging, with improved tolerability and higher rates of splenic volume reduction than was seen in the COMFORT studies [40]. HDAC inhibitors are recognized as being able to downregulate JAK2 activity via inhibition of HSP-90, an ATP-dependent molecular chaperone of a large number of proteins, including JAK2 [41]. JAK2 mutated cells have been shown to be more dependent on HSP-90 for preservation of their growth and survival than normal myeloid cells.

#### Ruxolitinib+ Bromodomain and extra-terminal (BET) inhibitor

Recent studies in MPN patients and in preclinical MPN models have shown that MPNs, in particular MF, are characterized by a chronic state of inflammation. In addition, increased levels of circulating cytokines are linked to adverse outcome in MF [42], consistent with a key role for inflammatory signaling in MPN progression and disease maintenance. These observations provide a strong rationale to investigate underlying generegulatory mechanisms that sustain chronic inflammation in MPNs. NF-kB acts as an important inflammatory signaling node in MPN. Bromodomain containing 4 (BRD4) has been shown to transcriptionally co-activate NF-kB through recognition and direct binding to acetylated p65 [43]. BET protein function is required for pathologic transcriptional NF-kB activity in MPN. In MPN mouse models, BET protein inhibition in combination with JAK kinase inhibition lead to complete reversal of reticulin fibrosis, reduce inflammatory signaling, reduce disease burden, and delays persistence associated with JAK inhibitors [44, 45]. These studies suggest that BET inhibition, particularly in combination with JAK kinase inhibition, should be evaluated for the ability to achieve substantive clinical benefit in MPNs patients.

#### Ruxolitinib+ poly-ADP-ribose polymerase (PARP) inhibitor

MPN cells contain elevated levels of reactive oxygen species (ROS) and stalled replication forks, resulting in accumulation of high numbers of toxic DNA double-strand breaks (DSBs) [46, 47]. Therefore, MPN cells survival may depend on DSB repair mechanisms [48]. PARP1 plays a central role in preventing/repairing lethal DSBs by activation of base excision repair/single-stranded DNA break repair, by stimulation of fork repair/restart, and by mediating the back-up NHEJ (B-NHEJ) repair [49]. Accumulation of potentially lethal DSBs in MPN cells could create opportunity to eliminate these cells by targeting DNA repair mechanisms.

Nieborowska-Skorska et al. [50] found that ruxolitinib inhibited two major DSB repair mechanisms, BRCA-mediated homologous recombination and DNA-dependent protein kinase–mediated nonhomologous end-joining, and, when combined with PARP inhibitor olaparib, caused abundant accumulation of toxic DSBs resulting in enhanced elimination of MPN primary cells, including the disease-initiating cells from the majority of patients. Moreover, the combination of Talazoparib, ruxolitinib, and hydroxyurea was highly effective in vivo in a*JAK2*V617F murine model and also against *JAK2*V617F, *CALR* (del52), and *MPL*W515L primary MPN xenografts. It is postulated that ruxolitinib-induced deficiencies in DSB repair pathways sensitized MPN cells to synthetic lethality triggered by PARP inhibitors.

### New targeting therapy

#### PRMT5 inhibitor

Liu et al. [37] found that JAK2V617F binds to PRMT5 with more affinity than does wild-type JAK2. These oncogenic kinases also acquired the ability to phosphorylate PRMT5, greatly impairing its ability to methylate its histone substrates, and representing a specific gain-of-function that allows them to regulate chromatin modifications. PRMT5 phosphorylation was readily detected in *JAK2*V617F-positive patient samples, and down-regulation of PRMT5 in human CD34+ cells resulted in increased colony formation and erythroid differentiation. These results indicate that phosphorylation of PRMT5 contributes to the mutant JAK2-induced myeloproliferative phenotype.

Sonderegger et al. [51] developed a potent and selective SAM-dependent inhibitor of PRMT5, CTx034. In vitro study, progenitor assays of bone marrow cells from patients with MPN showed that *JAK2*V617F erythropoiesis was more sensitive to CTx034 than normal erythropoiesis. In vivo studies showed normalization of spleen size and erythropoiesis, comparable to Ruxolitinib treatment. Importantly, CTx034 could eradicate the malignant clone, which is rarely achieved with Ruxolitinib. Moreover, CTx034 was well tolerated in healthy animals with no suppression of hematopoiesis. This result strengthens the therapeutic rationale for PRMT5 inhibitor in MPN.

#### Human double minute 2 (HDM2) inhibitors

HDM2 is an important negative regulator of p53 (promotes degradation of p53), and small-molecule inhibitors of HDM2 can trigger apoptosis in cells with intact p53 function by activating p53. Because IFN-a targets *JAK2*V617F progenitors in PV through activation of mitogen-activated protein kinase (MAPK) and STAT1, thereby increasing p53 transcription [52]. The combination of IFN-a with HDM2 inhibitors, which prevent the degradation of p53, provides an opportunity to induce p53-dependent apoptosis through disparate mechanisms. In vitro and in PDX mouse model [53, 54], HDM2 antagonist Nutlin-3 or HDM2 inhibitor RG7112, with pegylated IFN-a-2a significantly decreased MPN colony formation and preferentially eliminated *JAK2*V617F progenitors.

The clinical candidate HDM2 inhibitor idasanutlin is currently being studied in a phase 1 trial in patients with PV or ET (NCT02407080), with a provision for adding pegylated IFN-a-2a in subjects without at least a partial remission (PR) after 3 cycles. Of note, HDM2 inhibitors can cause significant thrombocytopenia by promoting p53-mediated apoptosis of megakaryocyte progenitors [55].

## Conclusions

The management of patients with MPN has been revolutionized by changes stemming from important discoveries regarding molecular pathogenesis, especially the central role of the JAK-STAT pathway. After the discovery of *JAK2*V617F and the demonstration that BCR-ABL-negative MPNs are driven by abnormal JAK2 activation, there were curative expectations for JAK inhibitors. Until now, type I ATP competitors targeting the ATP-pocket of the JH1 kinase, ruxolitinib, has remained the only approved therapy for MPNs. In particular, treatment for patients with MF and extensive splenomegaly and symptomatic burden has been significantly improved following the introduction of the ruxolitinib. However, ruxolitinib for MPNs is still largely inadequate to cope with significant challenges including reduction of mutated allele burden, reversion of fibrosis, normalization of life span and prevention of hematological progression [56]. Recently, some clinical trials of novel type I JAK inhibitors showed equal, even better effect of reduction in splenomegaly and transfusion and improvement in symptomatic burden and these results really shed light on treatment options for ruxolitinib-resistant or ruxolitinib-intolerant MPN patients. However, some of the major challenges of JAK2 inhibition: the first, to achieve potent catalytic inhibition without off-target effects; the second, to reach a high degree of selectivity for JAK2 against the other JAKs; the third, to specifically or preferentially target mutated forms against JAK2 WT, thus directly targeting the MPN clone and sparing normal hematopoiesis; finally, to specifically disrupt JAK2 function in defined cytokine receptor complexes were not overcame. Combinatorial approaches of ruxolitinib and various agents may lower toxicity and provide more effective disease control, yet more preclinical data are needed to define the most effective and synergistic combinations. In the coming decade, due to aforementioned disadvantages of ruxolitinib, the need for novel JAK inhibitors will be greater. The selectivity of JAK2, even JAK2 V617F could be achieved by developing allosteric inhibitors targeting sites outside of the active site. Allosteric inhibitors can present significant advantages compared to ATP-site binders. An allosteric site is defined as a region distinct from the kinase active site and where ligand binding can either positively or negatively regulate the enzyme activity [57]. This holds the potential of higher selectivity because every kinase possesses specific regulatory mechanisms. Moreover, targeting regions outside of the ATP-binding site will allow the development of compounds with different and improved physicochemical properties [58]. Another asset of allosteric inhibitors would be the possibility to overcome drug resistance by targeting two different sites in combination, as drug resistance usually emerges when mutations arise to prevent the compound from binding to one site [59].

**Fig. 1:**
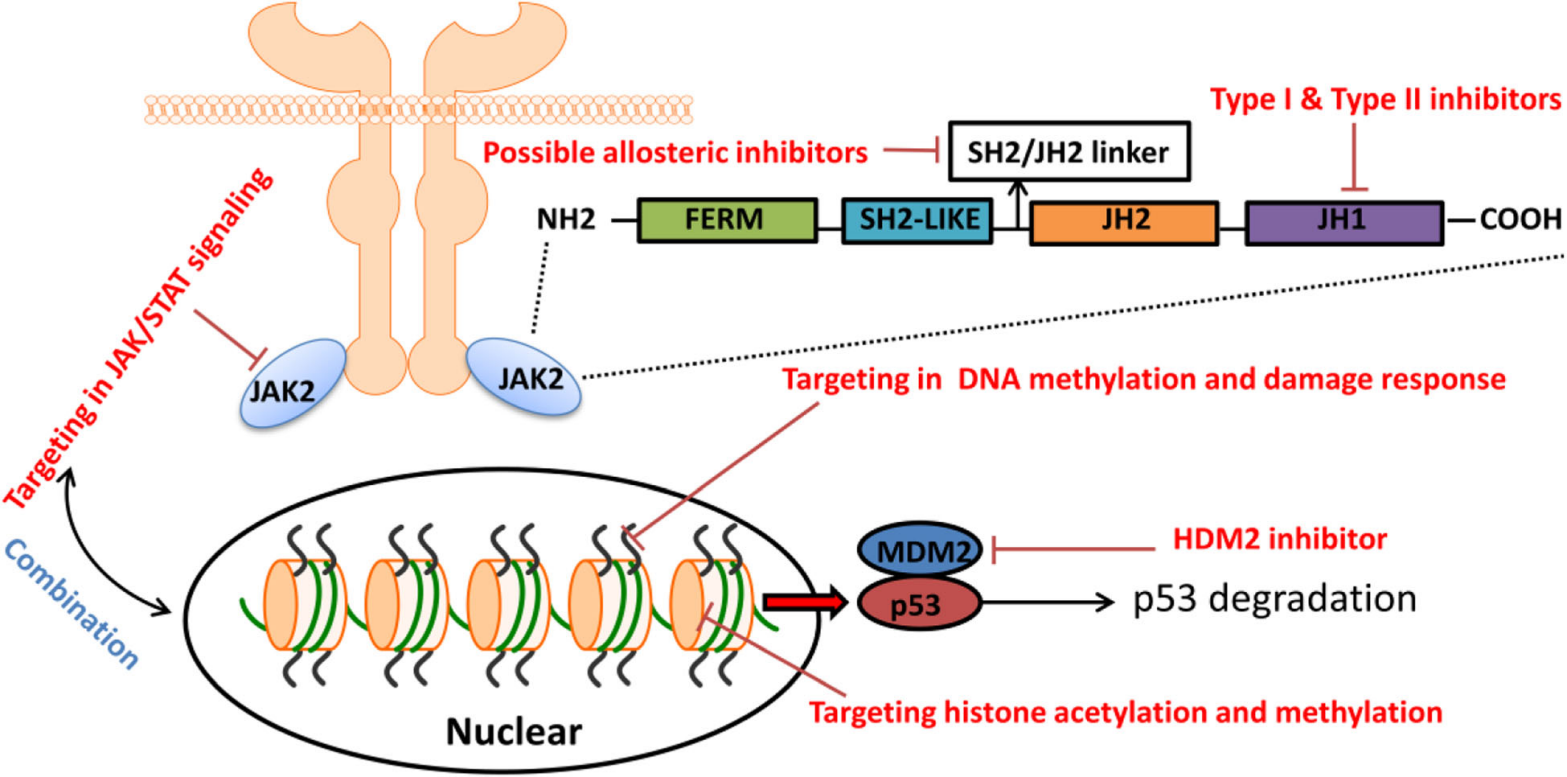
The common molecular targets in MPN treatment

Meanwhile, efforts should continue to identify other rational novel target drug targets, either alone or in combination with ruxolitinib on the basis of translational studies. Table 1 summarizes the undergoing clinical trials in MPN and Fig. 1 summarizes the common target in MPN treatment. Increased availability of next-generation sequencing and novel drugs targeting common gene mutations will go some way toward individualized targeting therapies in MPNs, especially for MF which has more complicated gene mutations than PV and ET.

**Table 1:** List of drugs undergoing clinical trials in MPN

NCT Number	Drugs	Target	Phase	Status
NCT03895112	AVID200	TGF-β1 and TGF-β3	Phase 1	Recruiting
NCT03566446	CALRLong 36 peptide	CALR	Phase 1	Active, not recruiting
NCT03144687	Itacitinib, Ruxolitinib	JAK	Phase 2	Recruiting
NCT03075826	SGI-110	DNMT	Phase 2	Recruiting
NCT03065400	Pembrolizumab	PD-1	Phase 2	Recruiting
NCT02718300	Parsaclisib, Ruxolitinib	PI3Kδ, JAK	Phase 2	Recruiting
NCT02493530	TGR-1202, Ruxolitinib	PI3Kδ, JAK	Phase 1	Recruiting
NCT02407080	RG7388, Pegasys	MDM2	Phase 1	Active, not recruiting
NCT02268253	SL-401	CD123	Phase1/2	Recruiting
NCT02257138	Decitabine, Ruxolitinib Phosphate	DNMT, JAK	Phase 1/2	Recruiting
NCT01761968	Givinostat	HDAC	Phase 2	Recruiting
NCT01633372	Itacitinib	JAK1	Phase 2	Active, not recruiting
NCT01594723	LY2784544	JAK	Phase 2	Active, not recruiting
NCT01393509	PU-H71	Hsp90	Phase 1	Active, not recruiting

## Abbreviations

ATP: Adenosine triphosphate
DNA: Deoxyribonucleic acid
FDA: Food and Drug Administration
HCT: Hematocrit
PDX: Patient-Derived Xenograft
